# Intraoperative electrophysiological evaluations of macular function during peripheral scleral indentation

**DOI:** 10.1038/srep35164

**Published:** 2016-10-20

**Authors:** Goichi Akiyama, Celso Soiti Matsumoto, Kei Shinoda, Gaku Terauchi, Harue Matsumoto, Emiko Watanabe, Takeshi Iwata, Atsushi Mizota, Yozo Miyake

**Affiliations:** 1 Department of Ophthalmology, Teikyo University School of Medicine, Kaga 2-11-1, Itabashi-ku, Tokyo 173-8605, Japan; 2 Matsumoto Eye Clinic, Tokushima, Japan; 3 Division of Molecular and Cellular Biology, National Institute of Sensory Organs National Hospital Organization Tokyo Medical Center, Tokyo, Japan; 4 Aichi Medical University, 1-1 Yazakokarimata, Nagakute, Aichi, 480-1195, Japan

## Abstract

Scleral indentation is widely used to examine the peripheral fundus, however it can increase the intraocular pressure (IOP) to high levels which can then affect retinal function. We evaluated the effects of scleral indentation on the macular function electrophysiologically. Intraoperative focal macular electroretinograms (iFMERGs) were recorded with and without controlling the IOP in 7 eyes. Without IOP control, the IOP increased from 21.7 ± 4.9 to 92.7 ± 20.2 mmHg significantly (*P* = 0.020) and the amplitudes of the b-wave (from 6.29 ± 1.160 to 3.71 ± 1.98 uV, *P* = 0.007), on-photopic negative response (from 2.29 ± 0.99 to 0.72 ± 0.47 uV, on-PhNR, *P* = 0.005), and d-wave (from 2.57 ± 0.41 to 1.64 ± 0.69 uV, *P* = 0.007) decreased significantly soon after beginning the indentation. All values returned to the baseline levels after releasing the indentation. In the eyes with IOP controlled, the IOP and the amplitude of all components did not change significantly during and after the indentation except the on-PhNR amplitude which was significantly reduced during the indentation. The changes in the iFMERGs and macular function caused by scleral indentation were transient and reversible. The changes can be minimized by controlling the IOP.

Scleral indentation is widely used during scleral buckling and vitreous surgeries^1^,^2^. Because considerable pressure is required to bring the peripheral retina and vitreous base into view, the eye is distorted and the intraocular pressure (IOP) is elevated to relatively high pressures^3^,^4^,^5^,^6^,^7^. Therefore, various adverse effects such as expulsive choroidal hemorrhage, vitreous hemorrhage, choroidal detachment, retinal ischemia, and ganglion cell damage^8^,^9^,^10^,^11^,^12^,^13^,^14^,^15^ can occur due to the increased IOP^16^. This increased IOP caused by scleral indentation needs to be kept in mind especially in more vulnerable eyes such as those with high myopia, glaucoma, and injured eyes.

Many investigators have examined the physiology of the retina following IOP elevation in animals^17^,^18^,^19^,^20^,^21^,^22^,^23^. These evaluations were done by full-field electroretinography (ERG), and in general the ERG components representing inner retinal function such as the scotopic threshold response (STR), oscillatory potentials, (OPs) and b-waves were found to be more sensitive to the IOP elevations. It is known that the IOP is elevated to high levels during scleral indentation, however the effect of scleral indentation on the local macular function has not been determined.

The technique for recording of focal macular electroretinograms (FMERGs) *in situ* was developed by Miyake *et al*. in 1981^24^. This technique can be used to evaluate a focal area of the macula in a way similar to that used to assess the physiological properties of a large area of the retina by full-field photopic ERGs. The diameter of the stimulus used to elicit the FMERGs was either 5°, 10°, or 15°, and it was delivered to a local retinal area while its position was continuously monitored with a fundus camera. The recorded FMERGs consisted of an a-, b-, and d-waves, the OPs, and the photopic negative response (PhNR). Since then, FMERGs have been used to assess the physiological condition of the different types of retinal neural cells including the photoreceptors in the macular area^25^,^26^,^27^,^28^,^29^,^30^.

Recently, a technique of recording FMERG during vitreous surgery or intraoperative FMERGs (iFMERG) has been developed^31^. However, this technique has not been used to determine the effect of scleral indentation on the iFMERGs.

Thus, the purpose of this study was to determine the effect of scleral indentation on the macular function. To accomplish this, we recorded iFMERGs combined with IOP measurements during scleral indentation in patients undergoing vitrectomy.

## Subjects and Methods

### Subjects

Intraoperative iFMERGs were elicited by focal stimulation of the macular area in 7 patients who were undergoing vitreous surgery with scleral indentation. There were 4 men and 3 women whose mean age was 70.1 ± 13.9 (±SD) years with a range of 43 to 87 years and a median of 69 years. The vitreoretinal pathologies were; 4 with an epiretinal membrane, 1 with a macular hole, and 2 with vitreous hemorrhage associated with diabetic retinopathy. The demographics of the patients are shown in Table 1. This study was conducted according to the tenets of the Declaration of Helsinki, and all procedures were approved by the Ethics Committee of Teikyo University School of Medicine. An informed consent was obtained from all subjects.

## Methods

### Preparation of patients

The eyes were anesthetized by a subtenon injection of 3 to 4 ml of lidocaine (2%), and the pupil was dilated with topical tropicamide (0.5%) and phenylephrine hydrochloride (5%). The non-stimulated eye was covered with the surgical drape.

### Vitreous surgery

Pars plana vitrectomy was performed with a conventional 3 port 25G vitrectomy system with valved trocars^32^. The temperature of the operating room was kept at 25 °C. The vitreous cutter was driven by a vitreous surgery system (Constellation Vision System^®^, Alcon Surgical, Fort Worth, Texas, USA). The intraocular pressure (IOP) was kept at 20 mmHg during the surgery with an IOP control system^16^ that was integrated into the system. The device included an infusion system that maintained the IOP constant. The infusion pressure at the console was increased to match the pressure drop across the cannula to maintain a constant IOP. The iFMERGs were also recorded with the IOP control system turned off, i.e., without IOP control.

**Figure 1:**
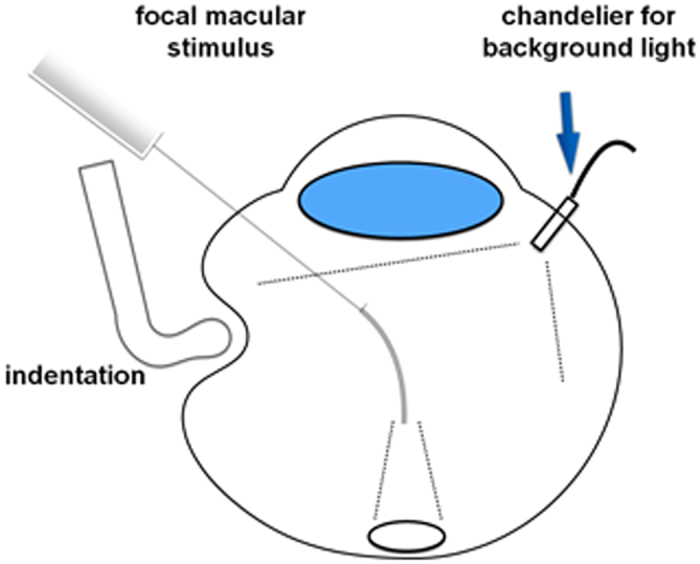
Drawing of the set up for recording intraoperative focal macular electroretinograms (iFMERGs] during scleral indentation. The focal macular stimulus light was delivered through a 25 gauge directional optic glass fiber so that the stimulus light illuminated the macula perpendicularly. The background light was provided by a chandelier type dual light fiber 29 G probe.

After core vitrectomy, iFMERGs were recorded before, during, and after scleral indentation with and without IOP control (Fig. 1). Scleral indentation was done by a single surgeon [CM] using a strabismus hook as routinely performed. The equator of the eye was indented gently until the indented retina could be seen through the wide viewing system of the operating microscope.

### Photopic Stimuli

An operating microscope (Model M844^®^, Leica Microsystems, Weltzer, Germany) combined with a wide angle fundus observation system (BIOM^®^, Oculus, Weltzer, Germany) was used for fundus observations during the focal retinal stimulation. White, high flux light emitting diodes (LED; OSW4XME3C1E, Optosupply, Taiwan) were used for the stimlus, and a spot stimulus was delivered to the macular area by a 25 G directional optic glass fiber (25 G Directional Laser Probe, Synergetics^TM^, MO, USA, Fig. 1). The directional probe was used to deliver the stimulation light to minimize the Stiles-Crawford effect (SCE)^33^. The size of the stimulus spot was controlled by the distance of the tip of the endoprobe from the retinal surface, and the probe was moved so that the size of the stimulus used was approximately 15°.

The iFMERGs were elicited by 5-Hz rectangular stimuli with a luminance of 270 cd/m^2^, duration of 100-ms light on and 150-ms light off, and background luminance of 3 cd/m^2^. The luminance of the stimulus was 1.95 log units greater than the surround luminance as in an earlier study^31^. The background illumination was used to depress the sensitivity of the area surrounding the focal stimulus which then reduced the contamination from the response elicited by stray light (Fig. 1). Because the focal stimulus was delivered directly onto the macula without passing through the ocular media, the stray light effect was minimized. The background light was obtained from a high flux LED light source and was diffusely spread on the retinal surface by a chandelier type dual light fiber 29 G probe (29ga, Oshima Dual Chandelier, Synergetics^TM^ Inc, MO, USA).

### Recording intraoperative focal macular electroretinograms (iFMERGs)

A gold foil monopolar contact lens electrode (Mayo Corporation, Nagoya, Japan) was sterilized and placed on the cornea of the examined eye to pick-up the iFMERGs. A silver plate reference electrode was placed on the forehead, and the ground electrode was attached to an ear lobe.

After centering and adjusting the stimulus size on the retinal area to be stimulated, the stimulation was begun to elicit the iFMERGs. The responses were amplified and averaged with a bioamplifier (MEB-9404, Nihon Kohden Corporation, Tokyo, Japan) and A/D converted at 16 bits (PCI-16/16UD, Contec, Japan). One hundred responses were averaged, and the sampling rate was 10 kHz. The responses were filtered to 20 to 200 Hz with a hardwired band pass filter for recording the a-, b-, and d-waves, and to 100 to 500 Hz to record the OPs. The noise level with the electrodes in place with no stimulus was less than 0.1 μV. All iFMERG recordings performed during surgery were done after 5 minutes of room light-adaptation. The illuminance at the patient’s cornea level was approximately 1000 lx.

### Intraocular pressure measurements

**Figure 2:**
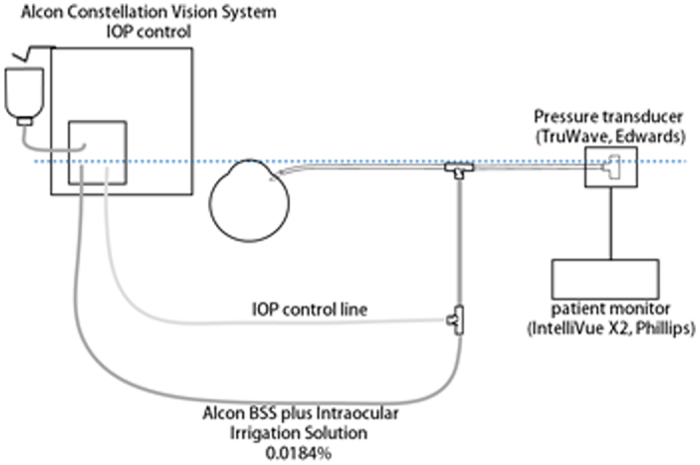
Drawing of the set up used to measure the intraocular pressure (IOP) during scleral indentation. The vented gas forced infusion (VGFI) system in the Constellation system controls the perfusion pressure by delivering the pressurized air into the bottle of balanced saline solution. A pressure transducer (TruWave) is connected to the infusion cannula through a side tube. The transducer is held at the level of the patient’s eye and the liquid level of the irrigation line in the cassette (dotted line). When the IOP control mode setting is not used, the irrigation tube was clamped between the transducer and the infusion bottle.

A drawing of an eye during the IOP measurements and scleral indentation is shown in Fig. 2. The IOP before, during, and after the scleral indentation was measured using a patient monitor (IntellivueX2^TM^, Koninklijke Philips N.V., Holland) and a disposable transducer (TruWave MP5100, Edwards Lifesciences Corporation, California, USA). This system allows monitoring the pressure of the fluid flow in the connecting cannula. Briefly, when the pressure in the monitoring tube is filled with balanced salt solution (BSS PLUS^®^ Irrigating Solution, Alcon Surgical, Fort Worth, Texas, USA) and is connected to a cannula inserted into the perfusion tube, the pressure is transmitted to the pressure transducer on the Pascal principle. Within the pressure transducer, the water and atmospheric pressure are separated by an elastic diaphragm. The diaphragm is bent by the difference between the perfusion pressure and the atmospheric pressure. A strain gauge attached to the atmospheric pressure side of the diaphragm can detect the deflection and converts it to an electrical signal. Then the signal is conveyed to the patient monitoring device and the irrigation pressure, i.e., the IOP, is continuously monitored. The transducer is connected to the infusion line, and the pressure of the flow at the connecting point is used as the real time IOP.

### Measurements of the different components of the intraoperative focal macular electroretinograms

The amplitudes and latencies of the a-, b-, and d-waves, and photopic negative responses (PhNRs) following the b-wave (on-PhNR), and the PhNRs following the d-wave (off-PhNR) were analyzed. The latency of the a-, b-, and d-waves, and the PhNR were measured from the stimulus onset to the peak of each wave. The amplitudes of the a-waves were measured from baseline to the trough, and the amplitude of the b-wave was measured from the trough of the a-wave to the peak of the b-wave. The amplitude of the PhNR was measured from the peaks of the b- or d-waves to the peak troughs of the on- or off-PhNR, respectively. The baseline was the potential at the time of the stimulus trigger. Because the trigger was delivered at 5 Hz and the response was continuously recorded for 200 msec, the end of each recording was the start of the next recording. The baseline was the potential at the beginning of the recording and it was same as the potential at the end of the recording.

All of the results are expressed as the means ± standard error of the means (SEMs).

Note: The procedures used conformed to the tenets of the Declaration of Helsinki. The study was a observational case series with approval of the Ethics Committee of the Teikyo University School of Medicine (Study ID Number: 10-033-2) and informed consent was obtained from all participants to participate in research.

## Results

**Figure 3:**
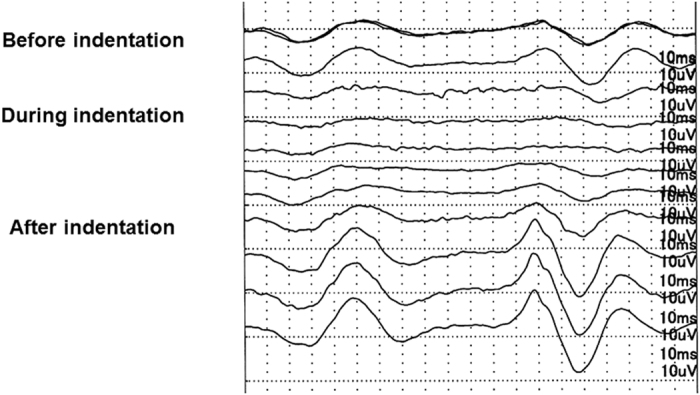
A representative case showing the continuous measurement of the intraoperative focal macular electroretinograms before, during, and after scleral indentation with the infusion cannula closed, i.e., without IOP control. Note that during the indentation the amplitude immediately decreased, and it recovered quickly after the indentation was released. The amplitudes at the recovery phase are significantly larger than that at the baseline.

All surgeries were performed successfully, and the visual acuity improved after the surgery in all eyes (Table 1). Representative data from one case with the IOP not controlled are shown in Fig. 3. In this case, the iFMERGs were recorded continuously before, during, and after the scleral indentation with the infusion cannula closed, i.e., with the IOP not controlled. Immediately after the scleral indentation began, the amplitudes of the different components of the iFMERGs decreased and almost disappeared. The iFMERG responses recovered quickly after the indentation was released. Interestingly, the amplitudes of the responses increased over the baseline values.

**Figure 4:**
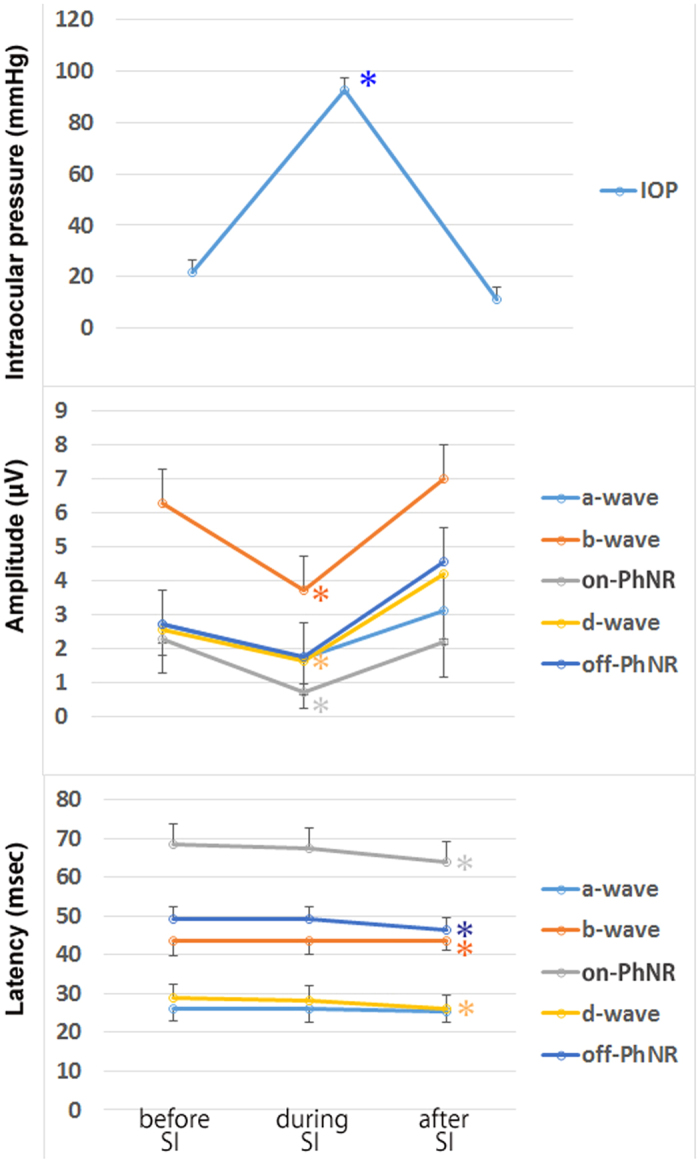
Intraocular pressures (upper), the amplitudes (middle), and the latencies (lower) of each component before, during, and after the scleral indentation when the IOP control was turned off. (Upper) The IOP was elevated during the scleral indentation and recovered to the baseline after releasing the indentation. (Middle) The amplitudes of the a-wave, on-PhNR, and d-wave were significantly reduced during the scleral indentation. Although it was not statistically significant, the amplitudes of the d-wave and the off-PNR were larger than that at the baseline after the indentation was released. (Lower) The latencies were stable during the indentation in all components. However, the latencies of the b-wave, PNR, and off PNR were significantly shorter after releasing the indentation compared to the baseline. IOP: intraocular pressure, PNR: photopic negative response, SI: scleral indentation.

Without IOP control, the IOP was elevated from 21.7 ± 4.9 to 92.7 ± 20.2 mmHg (*P* = 0.020) during the scleral indentation, and it returned to the baseline (11.0 ± 8.6 mmHg) after releasing the indentation (Table 2 and Fig. 4). The amplitude of each wave is shown in Tables 2 and 3. There was a relative large range of amplitudes of each component among the eyes. The amplitudes of the b-wave, on-PhNR, and d-wave were significantly reduced during the scleral indentation with the relative decrease in the amplitude different for the different components. After the indentation was released, the amplitudes of the d-wave and the off-PhNR were larger than that at the baseline. The overshoot was greater than the baseline amplitudes after the release of the indentation although it did not reach statistical significance.The latency was stable during the indentation in all components. However, the latencies of the b-wave, on-PhNR, and off-PhNR were significantly shorter after releasing the indentation compared to the baseline values (Table 2).

**Figure 5:**
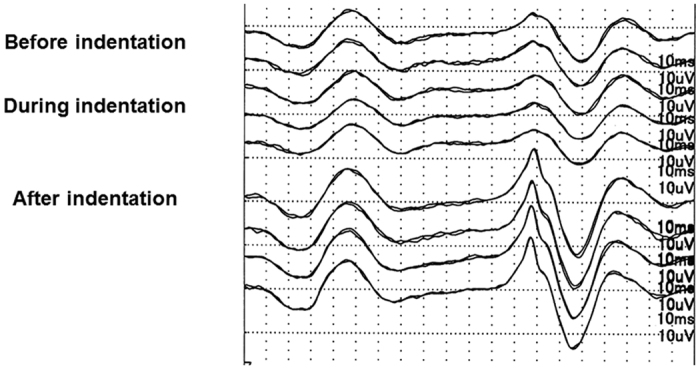
Representative case showing consecutive intraoperative focal macular electroretinograms before, during, and after the scleral indentation when the IOP control was tuned on. Note that during the indentation, the amplitude is not changed significantly.

**Figure 6:**
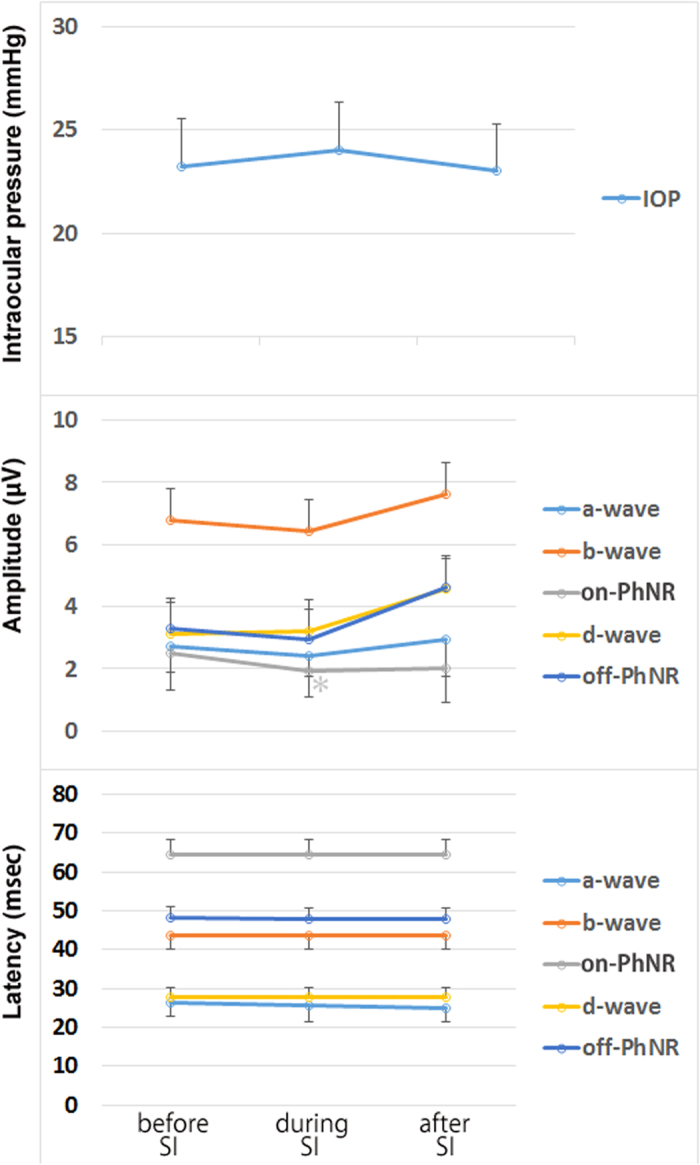
Intraocular pressures (upper), the amplitudes (middle), and latencies (lower) of the different components of the iFMERGs before, during, and after scleral indentation when the IOP control setting was turned on. (Upper) The IOPs do not change significantly during the scleral indentation. (Middle) The amplitudes of most components do not change significantly during the scleral indentation except the PNR was significantly reduced during the indentation to 77% of the baseline value, and the b-wave, d-wave, and the off-PNR overshot the baseline levels after the indentation. (Lower) The latencies of all components do not change significantly during and after the scleral indentation. IOP: intraocular pressure, PNR: photopic negative response, SI: scleral indentation.

Representative data from one case with the IOP controlled are shown in Fig. 5. With the IOP control functioning, the IOP was stable during and after the release of the indentation (Table 3 and Fig. 6). All components were not changed significantly throughout the scleral indentation procedure except the on-PhNR which was significantly reduced during the indentation to 63% of the baseline and the b-wave, d-wave and the off-PhNR had an overshoot after the indentation. On the other hand, the latency was stable during and after releasing the indentation for all components (Table 3).

## Discussion

Our results showed that the IOP was increased and the iFMERGs were reduced during the scleral indentation when the IOP-control system was turned off. The depression of the macular function in conjunction with the IOP elevation can be explained by an impairment of ocular circulation. Yancey *et al*.^17^ investigated the effect of IOP elevation on the full-field ERGs and the choroidal oxygen tension (PO_2_) under dark-adapted conditions in feline eyes. They reported that the choroidal PO_2_ and the b-wave amplitude were decreased with increased IOP. Tazawa *et al*.^34^ recorded full-field ERGs in the living extracorporeal bovine eyes, and reported that the b-wave was the first component to decrease and the a-wave were later decreased under anoxic conditions. With re-perfusion, the a-waves were the first to recover followed by the b-waves. The current results are in good agreement with their results.

There are three new findings in our results. First, our data showed that the on-PhNR and the d-wave were also altered during the IOP increase. Animal experiments^18^,^20^,^22^ have shown that the STR and OPs, which originate from the activity of the neural cells in the inner retinal layer, were more sensitive than the cells contributing to the a- and b-waves. The PhNR and the STR are believed to reflect the activity of the inner retinal layer especially the retinal ganglion cells, and our data agree well with the results of the earlier animal data. Although careful interpretation should be made because our ERGs were elicited by a focal macular area and the IOP changes were different than that used in the animal experiments. However, these results suggest that ganglion cells are the cells most susceptible to the IOP elevation irrespective of scotopic or photopic conditions. This explains the clinical findings that eyes with central retinal artery occlusion often show severe visual field loss despite a relatively well preserved b-wave. In such cases, the ganglion cells were severely damaged and responsible for the visual field loss^35^.

Second, the results showed that the all components immediately recovered with the return of the IOP to the baseline values, and the a- and b-waves and off responses overshot the baseline potential. This suggests that hyperperfusion might have taken place just after releasing the scleral indentation. In retinal ischemia-reperfusion experiments on cats, it was reported that within 5 minutes of the return of ocular circulation, the retinal blood flow was approximately 200% of the baseline level, and the choroidal blood flow was 108% of the baseline level^36^. Tazawa *et al*.^34^ also reported that the a-waves alone had an overshot during the recovery of perfusion in bovine eye in mixed rod and cone ERGs. They observed an overshoot of the rod component, while we observed an overshoot of the photopic component which mainly originates from the neural activity of the neurons in the inner retinal layer. In addition, an increase in the PhNR amplitude has been reported following the lowering of the IOP in eyes with glaucoma and ocular hypertension^37^. The authors suggested that this was due to improved glial function perhaps by a more effective buffering of the potassium concentration which led to inducing larger currents of the glial cells. It has been reported^38^ that the incidence of type-I retinopathy (positive demarcation line) was lower in low birthweight infants who underwent fundus examination using scleral indentation and on whom ocular massage was applied, compared to the low birthweight infants who underwent fundus examination without using scleral indentation and no pressure stimulation was administered to the globe. Our results support their findings in that the transient ocular pressure elevation and reduction may enhance retinal activity which has some good effect on the retina.

Third, the latency was stable during the indentation but shortened after the release of the indentation in the most components. This might be explained by the hyperperfusion. Interestingly, the latency tended to be changed in response to the IOP decrease compared to the amplitude, although it was the opposite in response to the IOP increase. The ERG response to the acute decrease of abnormally elevated IOP such as by the release of scleral indentation or the treatment of an acute glaucoma attack, or just after undergoing vacuum trephine during laser *in situ* keratomileusis (LASIK)^39^ is not completely understood. Further investigations are needed to clarify the ERG responses when the abnormally elevated IOP returns to normal levels.

Scleral indentation can be performed on an eye with vitreous such as during scleral buckling surgery and peripheral retinal examination in an outpatient clinic. Under such conditions, the IOP can be increased much higher, e.g., 116–350 mmHg^4^,^5^, than that in this study. Although the IOP elevation depends on various factors such as scleral resistance, the depression technique, and the location of depression^3^, it should be noted that we observed an average IOP elevation to 92.7 mmHg when the IOP control was turned off. The valved trocars used during modern vitreous surgery have decreased the risk of hypotony during scleral indentation in air-filled eye. On the other hand, the IOP could increase to considerable levels when the vitreous cutter or extrusion needle is not functioning such as during peripheral examination or endo-laser photocoagulation. However, we observed a minimal change in the IOP and ERG responses under the IOP control setting. Sugiura *et al*.^16^ showed that it required only a few milliseconds to stabilize IOP during vitrectomy under the IOP control setting. Although our results showed that the functional changes caused by scleral indentation were transient and reversible, we recommend that the degree of elevation should be minimized by judicious manipulation and the IOP control system especially on eyes with glaucoma or circulatory disturbances and the eyes of children^20^,^21^,^40^,^41^.

ERG monitoring during human eye surgery was first reported in 1991^42^,^43^. The investigators evaluated the mass retinal response with full-field ERGs using a contact lens electrode integrated with a light-emitted diode during eye surgery^42^,^43^,^44^. No changes were observed during the extraocular manipulation such as conjunctival preparation and extraocular muscle manipulation but significant changes were observed during scleral buckling and vitreous removal. The vitreous removal led to a considerable decrease in the amplitude and prolongation of the peak time in the flicker ERG responses. These ERG changes were reversible and associated with the temperature of the irrigation solution. They also reported a reversible reduction and even a complete loss of the flicker response with an elevation of the irrigation pressure to 75 mmHg^44^. Although the iFMERGs can be performed only during vitreous surgery, it enables an *in situ* assessment of the macular function, and we were able to examine the effects of the scleral indentation during surgery.

There are limitations in this study. One limitation is the small number of eyes studied and the different diseases of the patients. Thus, the results should be interpreted with caution. A relatively large variation in the amplitudes might limit the statistical power of the data. Repeated measurements were not performed because the treatment of the patient’s condition comes first. Although the vitreoretinal pathology varied, eyes with severe macular disease were not included in this study, and good iFMERG recordings and analysis on them are possible. Studies with a large number of subjects are required to confirm our findings.

Another limitation was that the degree, speed, and strength of the indentation depend on the manipulations of the surgeons and might be inconsistent. However, the manipulation was performed as routine procedure and the IOP change was within the range reported in the previous investigations^3^,^16^, therefore we believe that the scleral indentation maneuver well reflects the widely performed procedure in a clinical situation.

In conclusion, the iFMERG can inform us on the physiological condition of the macula during vitreous surgery when the retina is exposed to un-physiological conditions. The iFMERG would be useful to assess the influence of the several surgical procedures.

## Additional Information

**How to cite this article**: Akiyama, G. *et al*. Intraoperative electrophysiological evaluations of macular function during peripheral scleral indentation. *Sci. Rep.* **6**, 35164; doi: 10.1038/srep35164 (2016).

## Figures and Tables

**Table 1:** Patients’ demographics.

Case	Age	R or L	vitreoretinal pathology	Best corrected visual acuity	
				pre-operation	post-operation
1	66	L	VH PDR	0.1	1.2
2	85	R	ERM	0.4	0.5
3	77	R	ERM	0.1	0.4
4	87	L	ERM	0.4	0.8
5	69	R	ERM	0.8	1
6	64	R	MH	0.1	0.6
7	43	L	VH PDR	0.01	0.8

L: left, R: right, VH: vitreous hemorrhage, PDR: proliferative diabetic retinopathy.

ERM: epiretinal membrane, MH: macular hole.

**Table 2:** The amplitude and latency before, during, and after scleral indentation (SI) without IOP control setting.

		a-wave		b-wave	p value^#^	on-PhNR	p value^#^	d-wave	p value^#^	off-PhNR	p value^#^
**Amplitude (uV)**											
before SI	mean ± SD	2.71 ± 0.92		6.29 ± 1.160		2.29 ± 0.99		2.57 ± 0.41		2.71 ± 0.99	
(baseline)	range	1.5~4.0		4.0~8.0		1.0~4.0		2.0~3.0		1.5~4.0	
during SI	mean ± SD	1.71 ± 106	0.105	3.71 ± 1.98	0.007*	0.72 ± 0.47	0.005*	1.64 ± 0.69	0.007*	1.78 ± 0.99	0.162
	range	0.0~3.0		0.0~6.0		0.0~1.5		0.0~2.0		0.0~3.0	
after SI	mean ± SD	3.14 ± 1.03	0.2	7.00 ± 2.98	0.527	2.21 ± 1.06	0.89	4.21 ± 1.94	0.09	4.57 ± 2.37	0.054
	range	1.5~4.0		2.0~11.0		1.0~4.0		2.0~8.0		2.5~10.0	
**Latency (msec)**											
before SI	mean ± SD	26.00 ± 2.73		46.71 ± 3.84		68.57 ± 5.15		28.86 ± 3.68		49.29 ± 3.19	
(baseline)	range	22.0~30.0		40.0~52.0		60.0~75.0		25.0~35.0		45.0~55.0	
during SI^†^	mean ± SD	26.17 ± 2.91	1^†^	45.83 ± 3.44	1^†^	67.50 ± 4.79	1^†^	28.33 ± 3.73	1^†^	49.17 ± 3.44	1^†^
	range	22.0~30.0		40.0~50.0		60.0~75.0		25.0~35.0		45.0~50.0	
after SI	mean ± SD	25.29 ± 2.36	0.604	43.86 ± 2.47	0.012*	64.00 ± 3.82	0.008*	26.14 ± 1.36	0.05*	46.29 ± 2.66	0.01*
	range	20.0~30.0		40.0~48.0		60.0~70.0		25.0~28.0		42.0~50.0	

IOP: intraocular pressure, PhNR: photopic negative response, SD: standard deviation.

^#^Statistical comparison by paired t-test compared to baseline, *significant difference compared to baseline (p < 0.05).

^†^The latency was unmeasurable in one case who showed no response during indentation.

Therefore the paierd t-test was performed in the rest 6 cases.

**Table 3:** The amplitude and latency before, during, and after scleral indentation (SI) with IOP control setting.

		a-wave		b-wave	p value^#^	on-PhNR	p value^#^	d-wave	p value^#^	off-PhNR	p value^#^
**Amplitude (uV)**											
before SI	mean ± SD	2.71 ± 0.84		6.79 ± 1.85		2.50 ± 1.20		3.14 ± 0.99		3.28 ± 0.84	
(baseline)	range	1.5~4.0		3.5~9.0		1.0~4.5		2.0~5.0		2.0~4.5	
during SI	mean ± SD	2.43 ± 0.68	0.356	6.43 ± 1.76	0.182	1.93 ± 0.82	0.030*	3.21 ± 0.88	0.788	2.93 ± 0.94	0.094
	range	1.5~3.5		4.0~9.0		1.0~3.5		2.0~4.5		1.0~4.0	
after SI	mean ± SD	2.93 ± 1.18	0.589	7.64 ± 2.49	0.053	2.00 ± 1.10	0.086	4.57 ± 3.16	0.245	4.64 ± 3.21	0.278
	range	1.5~4.5		4.0~11.0		1.0~4.5		2.0~12.0		2.0~12.0	
**Latency (msec)**											
before SI	mean ± SD	26.43 ± 3.50		43.57 ± 3.50		64.29 ± 4.16		27.86 ± 2.47		46.86 ± 2.16	
(baseline)	range	20.0~30.0		40.0~50.0		60.0~70.0		25.0~30.0		45.0~50.0	
during SI	mean ± SD	25.71 ± 4.16	0.356	43.57 ± 3.50	1	64.29 ± 4.16	1	27.86 ± 2.47	1	46.86 ± 2.16	1
	range	20.0~30.0		40.0~50.0		60.0~70.0		25.0~30.0		45.0~50.0	
after SI	mean ± SD	25.00 ± 3.78	0.172	43.57 ± 3.50	1	64.29 ± 4.16	1	27.86 ± 2.47	1	46.86 ± 2.16	1
	range	20.0~30.0		40.0~50.0		60.0~70.0		25.0~30.0		45.0~50.0	

IOP: intraocular pressure, PhNR: photopic negative response, SD: standard deviation.

^#^Statistical comparison by paired t-test compared to baseline, *significant difference compared to baseline (p < 0.05).
